# Advances in the Astonishing World of Phytochemicals: State-of-the-Art for Antioxidants

**DOI:** 10.3390/antiox12081581

**Published:** 2023-08-08

**Authors:** Marianna Lauricella, Antonella D’Anneo

**Affiliations:** 1 Department of Biomedicine, Neurosciences and Advanced Diagnostics (BIND), Institute of Biochemistry, University of Palermo, 90127 Palermo, Italy; 2 Laboratory of Biochemistry, Department of Biological, Chemical and Pharmaceutical Sciences and Technologies (STEBICEF), University of Palermo, 90127 Palermo, Italy

In recent years, research on phytochemicals has underscored pleiotropic actions and medicinal and health-promoting properties which certainly deserve serious attention. Natural-derived molecules, such as phytohormones, glycosides, terpenoids, alkaloids, and phenolic compounds, offer a protective or preventative shield against many several pathological conditions such as aging, cardiovascular diseases, diabetes, obesity, cancer, asthma, and neurodegenerative disorders [1,2]. On the other hand, the multi-faceted potentials of phytocompounds isolated from different parts of plants or fruits stimulate the interest of the pharmaceutical, nutraceutical, and cosmetic industries. A main goal of these companies is to identify new and innovative phytomolecules to use as they are as natural reservoirs in plants or to appropriately modify them with the insertion of pharmacophore groups to design enhanced derivatives [3].

In the light of these considerations, we put together this Special Issue, titled “Advances in the Astonishing World of Phytochemicals: State-of-the-Art for Antioxidants”, containing seventeen papers (fourteen research articles, one review, one comment, and a reply).

The scientific evidence reported in the SI analyzed both distribution and pleiotropic beneficial effects (antidiabetic, antitumor, antiflogistic, antibacterial, etc.) of some bioactive compounds with antioxidant properties. However, it also has to be considered that the relative abundance as well as the distribution of phytochemicals in plants or fruits is consistently affected by different parameters, such as environmental edaphic conditions, ripeness degree of fruits, and right season harvest [4].

In their contribution, Ali et al., for example, demonstrated that Australian fruits and spices such as mountain pepper berries (*Tasmannia lanceolata*), rosella (*Hibiscus sabdariffa*), lemon aspen (*Acronychia acidula*), and strawberry gum (*Eucalyptus olida*) represent a rich reservoir of bioactive phenolic metabolites (phenolic acids, flavonoids, isoflavonoids, tannins, stilbenes, lignans, and limonoids). Among these, the analysis provided evidence that both *Eucalyptus olida* and *Tasmannia lanceolata* possess the highest antioxidant and antidiabetic potential [5], a property that could be exploited in the development of specific biopharmaceuticals.

The effect of environmental conditions on the content and quality of phytochemicals was recently reported in studies performed on Amaranth, a leafy vegetable capable of growing under several salinity and drought-stress-induced conditions [6]. Salt stress has been demonstrated to enrich the amount of bioactive compounds with antioxidant properties. Indeed, the application of salt eustress conditions (25–100 mM NaCl) was able to boost the profile of microelements, macro-elements, phytochemicals, and phenolic acids in *Amaranthus gangeticus*, contributing to providing excellent quality in the end product for its antioxidant properties [7].

Studies performed on Romanian *Armoracia rusticana L*., a horseradish plant widely appreciated for its medicinal and aromatic properties, offered a complete profile of the low-molecular-weight metabolites of the plant grown in Romania. Nine categories of secondary metabolites (glucosilates, fatty acids, isothiocyanates, amino acids, phenolic acids, flavonoids, terpenoids, coumarins, and miscellaneous) were identified, and the development of phyto-engineered carrier systems capable of merging the biological properties of horseradish and kaolinite was proposed [8]. As a whole, the conclusion is that these systems could represent a possible controlled drug release system to apply to cancer-specific targeting.

In another study, Rani et al. explored the biological potential of dichloromethane and methanol root and shoot extracts of *Dryopteris juxtapostia*, a species belonging to the *Dryopteris* genus growing in the states of the north temperate zone. The study demonstrated that both extracts exerted radical-scavenging and anti-inflammatory and antitumoral effects in vitro as well as hepatoprotective actions in rats. *D. juxtapostia* root dichloromethane extracts exhibited the highest biological potential compared to other extracts, thus demonstrating the importance of using dichloromethane to obtain extracts enriched in phenolic components [9].

In addition, Vieira et al. demonstrated the anti-inflammatory effects of roots and flowers extracts of *E. purpurea*, a plant whose extracts are traditionally used to treat cold and flu. The study compared the effects of dichloromethanolic and ethanolic root and flowers extracts with alkylamide-rich extracts obtained by using the accelerated solvent extractor system, a green and innovative extraction technique. The authors concluded that all the extracts were capable of reducing the IL-6 levels as well as the intracellular levels of ROS/RNS in lipopolysaccharide-stimulated human-monocyte-derived macrophages. However, the alkylamide fractions possessed the strongest anti-inflammatory effects, thus evidencing these compounds as the main active extract components [10]. 

A fruit particularly rich in phytocompounds is tomato (*Lycopersicon esculentum* Mill.), a food largely consumed for its nutritive and nutraceutical properties [11]. Noteworthy, the different phytonutrient composition and antioxidant properties of the tomato are related to the different ripening times. On these bases, the study of Gambino et al. compared the different phytonutrients composition and properties of golden tomato (GT), a food product harvested at an incomplete ripening stage with respect to red tomato (RT), harvested at full maturation [12]. The authors demonstrated that GT contains a higher level of naringenin and chlorogenic acid, two polyphenols with antilipemic effects [13,14], than RT. Regarding biological activities, GT displays a better reducing power compared to RT [15]. Interestingly, GT oral supplementation in high-fed rats reduced body-weight gain and LDL cholesterol levels, as well as lowered oxidative stress markers both in the blood and liver, thus suggesting a potential of “GT” oral supplementation.

The biological properties of *Urtica dioica* (UD), *Matricaria chamomilla* (MC), and *Murraya koenigii* (MK), traditionally used in Ayurvedic medicine as nerve relaxants and cognition enhancers [16], were evaluated in the study of Shabir et al. [17]. Considering the effects of these plants on the nervous system, the authors investigated the ability of aqueous and ethanolic extracts of UD, MC, and MK to ameliorate the toxic effects of rotenone, a neurotoxic natural pesticide, in wild-type *Drosophila melanogaster*. The study evidenced the ability of plant extracts to exert neuroprotective effects on *Drosophila melanogaster* by alleviating rotenone-induced oxidative stress, enhancing locomotion, and restoring acetylcholine levels, thus suggesting a potential use of these extracts to treat neurological diseases. Of course, the right recovery of phytochemicals also depends either on the type of extraction techniques or solvents applied in the extraction procedure. This aspect was clearly addressed by Boyadzhieva et al., demonstrating a good recovery efficiency of phytochemicals from different parts (leaves, flowers, and stems) of *Gnaphalium viscosum* (Kunth, such as the antioxidants kaempferol, kaempferol-3-O-β-d-glucoside, and chlorogenic acid). Interestingly, for the first time, this study also demonstrated the presence in this species of leontopodic acids A and B, two highly potent antioxidants derived from glucaric acid [18].

In a study performed in yarrow (*Achillea millefolium L*.), a flowering plant commonly used in folk medicine to alleviate symptoms related to gastrointestinal discomfort [19], Villalva at al. used a supercritical antisolvent fractionation process [20] to obtain two different fractions containing polar phenolic compounds and monoterpenes and sesquiterpenes, respectively. Both the fractions explained the antibacterial effects observed against *Helicobacter pylori* strains. Furthermore, the extracted fractions exerted antioxidant and anti-inflammatory effects in *Helicobacter pylori*-infected human gastric AGS cells. From this study, we can conclude that yarrow extracts can be useful against *Helicobacter pylori* infection. The Villalva’s data have been criticized by Franski and Beszterda-Buszczak [21]. Although they do not question the quality of the paper, these authors raised questions about the correctness of some compounds identified by Villalva et al. However, Villalva clarified all the doubts of Franski and Beszterda-Buszczak in a reply paper [22].

Nowadays, there is great interest in the bio-waste products of agriculture for the presence of bioactive healthy compounds [23,24]. Thinning young apples (TAPs) are usually discarded to guarantee the output and to increase the quality of harvested apples. However, it has been shown that TAPs contain more than 10-fold polyphenols with respect to harvested apples [25]. In their contribution, Ferrario et al. characterized the profile of polyphenols in TAP using a dual LC-HRMS metabolomic approach to identify a total of 68 polyphenols. According to this investigation, TAP fractions exert both antioxidant and anti-inflammatory effects by up-regulating the nuclear-factor-erythroid-2-related factor (Nrf2) signaling pathways and inhibiting NF-kB activation in cell models [26]. These results evidenced TAP as a source of bioactive molecules endowed with antioxidant properties.

The presence of bioactive compounds has also been Identified in marine environments. For example, seaweeds, such as red (*Rhodophyta*), green (*Chlorophyta*), and brown algae (*Phaeophyta*), which are not included in the diet of the Western world, are widely spread in Asian and Chinese nutrition for their high-quality profile in bioactive molecules as phenolic compounds, vitamins, pigments, and essential minerals. The use of a green pressured liquid extraction technique allowed Perez-Vazquez et al., under specific experimental conditions of temperature, type of used solvent, extraction time, and pressure, to recover a high yield of active biomolecules to exploit on both a pharmaceutical and food industrial scale [27].

Notably, a recent study of Liberti et al. demonstrated the antioxidant and anti-inflammatory properties of sulfated exopolysaccharides (s-EPSs) and phycoerythrin (PE), two molecules naturally produced by the red marine microalga *Porphyridium cruentum*. In particular, s-EPSs were able to prevent GSH depletion and lipid peroxidation on a cell-based system but not in vitro, while PE showed high ROS scavenging capacity both in vitro and on a cell-based system. Interestingly, both the compounds were capable of inhibiting the pro-inflammatory enzyme COX-2 and promoting a fast scratch closure [28]. Altogether, the data obtained support the use of these compounds isolated by *P. cruentum* as anti-inflammatory components of medical patches.

The identification of phytomolecules with potential tailored applications represents a significant goal in the phytochemistry field. Particularly significant is the research discussed by Notaro et al. exploring the biochemical action of methyl gallate (MG), a gallotannin widely used in traditional Chinese phytotherapy to alleviate several cancer symptoms [29]. The findings reported by the authors shed light on the antitumor potential of MG. This phytocompound preferentially targeted HCT116 colon cancer cells, with respect to differentiated Caco-2 cells, an enterocyte-like cell model. In colon cancer cells, MG induced an oxidative injury sustained by ROS generation and endoplasmic reticulum stress as well as an upregulation in intracellular calcium content. In the first phase of treatment, oxidative events were accompanied by an autophagic process, that, for longer times of incubation, culminated in the apoptotic cell demise with DNA fragmentation and p53 and H2Ax activation. A particular role in the MG-induced mechanism was played by the oncosuppressor p53 protein. The conclusion of this research revealed the existence of an intertwined relationship between oxidative stress and p53 as a causative event in apoptotic cell death. Such a study paves the way to future investigations of MG alone or in combination treatment as a preventative or adjuvant phytocompound to apply in colon cancer treatment.

However, beyond these effects, bioactive compounds present in plants have also been demonstrated to play a protective role against oxidative injury, an aspect recently studied by Lv et al. in Caco-2 cells. The use of proanthocyanidins purified from kiwi leaves (*Actinidia chinensis*) counteracted both H_2_O_2_-induced oxidative damage as well as malondialdehyde increase [30]. Such an effect was accompanied with an upregulation of antioxidant systems (GSH-px, CAT, T-SOD) and the corresponding mRNA targets of Nrf2, the master regulator of the cellular stress response [31]. The conclusion of this interesting study is that the characterization of the antioxidant properties of kiwi leaves proanthocyanidins emphasizes their possible functional application either for a policy of circular economy or for sustainable industrial use.

The whole antioxidant activity of a sample cannot be ascribed only to a single bioactive component, but in many cases the overall potential is the result of the combinatorial effect of more components, acting in a synergistic, antagonistic, or additive manner. The comparative analysis of 10 phenolic acids (protocatechuic, gentisic, gallic, vanillic, syringic, p-coumaric, caffeic, ferulic, sinapic, and rosmarinic acid) used alone and in different combination mixtures provided evidence of the high antioxidant activity of gallic acid by a ferric reducing antioxidant power (FRAP) technique and a good oxygen radical absorbance capacity of rosmarinic acid by ORAC assays [32]. A relevant aspect of this study relied on the observation that hydroxybenzoic acid mixtures containing gentisic acid showed a clear synergistic action. These data strongly sustain the idea that the biological activity of a mixture, in some cases, cannot be ascribed to a single compound, but it has to be searched in the combination of compounds present and their ability to interact with each other.

We would like to share our gratitude to all authors who submitted their outstanding research to this Special Issue. Their manuscripts highlighted the role of natural-derived compounds with antioxidant potential action to apply as preventative or adjuvant molecules in the treatment of some chronic human diseases. Additionally, the identification of extraction techniques and solvents that can maximize the extraction of bioactive compounds is of great importance.
